# *Calcineurin Aβ* gene knockdown inhibits transient outward potassium current ion channel remodeling in hypertrophic ventricular myocyte

**DOI:** 10.1515/biol-2021-0107

**Published:** 2021-09-21

**Authors:** Long Yang, Na Deng, Jionghong He, Guiling Xia, Ying Yang, Yidong Zhao, Zhaomei Huo, Chuxian Guo

**Affiliations:** Department of Cardiology, Guizhou Provincial People’s Hospital, No. 83 Zhongshandong Road, Guiyang 550002, China; Department of Cardiology, The Affiliated Hospital of Guizhou Medical University, Guiyang 550025, China

**Keywords:** ventricular hypertrophy, transient outward potassium current, calcineurin, ion channel remodeling, action potential

## Abstract

It has been shown that the activation of calcineurin is involved in regulating ion channel remodeling in hypertrophic cardiomyocytes. But the precise role of calcineurin in the regulation of transient outward potassium current (*I*_to_), an ion channel associated with fatal arrhythmia, remains controversial. This study aimed to examine the effects of *calcineurin Aβ* (*CnAβ*) gene knockdown on *I*_to_ channel remodeling and action potential duration (APD) in the hypertrophic ventricular myocytes of neonatal rats. Results showed that phenylephrine stimulation caused hypertrophy of ventricular myocytes, upregulation of CnAβ protein expression, downregulation of *Kv4.2* mRNA and protein expression, a decrease in *I*_to_ current density, and prolongation of APD. *CnAβ* gene knockdown significantly inhibited the effects of phenylephrine stimulation. Our data indicate that *CnAβ* gene knockdown can inhibit *I*_to_ channel remodeling and APD prolongation in hypertrophic neonatal rat ventricular myocytes. This finding suggests that calcineurin may be a potential target for the prevention of malignant ventricular arrhythmia in a hypertrophic heart.

## 1 Introduction

Ventricular remodeling caused by pathological cardiac hypertrophy is chronic congestive heart failure’s most important pathophysiological mechanism [1,2]. Sudden cardiac death is the dominant reason of death in patients with congestive heart failure [3]. Ion channel remodeling in ventricular myocytes is the main pathophysiological basis leading to changes in the action potential duration (APD) of ventricular myocytes, resulting in malignant ventricular arrhythmia [4,5]. Congenitally, dysregulation of transient outward potassium current (*I*_to_) has also been demonstrated to play a pivotal role in the Brugada syndrome [6,7].

*I*_to_ is a rapidly activated and inactivated outward potassium current, mainly involved in phase 1 of action potentials. The activity of *I*_to_ channels influences the activation of voltage-gated Ca^2+^ channels and the balance of inward and outward currents during the plateau, thereby mediating the duration and the amplitude of phase 2. In cultured hypertrophic ventricular myocytes, or the ventricular myocytes from a myocardial infarction heart, downregulated or dysfunctional *I*_to_ channels of ventricular myocytes lead to delayed repolarization and prolonged APD, which may easily cause fatal arrhythmia [8,9].

Calcineurin, a Ca^2+^-calmodulin-regulated phosphatase, has been shown to participate in hypertrophic signal transduction. Research have shown that the continuous activation of calcineurin promotes cardiac hypertrophic remodeling, decompensated heart failure, and arrhythmic death [10,11]. Additionally, calcineurin regulates ion channel remodeling in hypertrophic cardiomyocytes [9,12,13,14,15].

In transgenic mice, overexpression of calcineurin results in cardiac hypertrophy and the downregulation of *I*_to_, which was reversed by the calcineurin inhibitor cyclosporine [13]. In rats after myocardial infarction, cyclosporine significantly attenuated the decreases in mRNA levels of Kv4.2 and Kv4.3, the components of α subunit in *I*_to_ channel, and *I*_to_ density in the left ventricular [14]. In cultured adult canine left ventricular cardiomyocytes, rapid pacing reduced *I*_to_ density and Kv4.3 mRNA and protein expression, which was markedly prevented by inhibiting calcineurin with cyclosporine [15]. Those results indicated that the activation of calcineurin may lead to *I*_to_ downregulation. Conversely, there was also evidence in cultured neonatal rat ventricular myocytes that the overexpression of constitutive calcineurin upregulates Kv4.2 expression without affecting Kv4.3 [9]. Thus, the precise role of calcineurin in the regulation of *I*_to_ remains unclear.

The purpose of this study is to clarify the regulatory effect of calcineurin on *I*_to_ channel remodeling and APD alterations in the hypertrophic ventricular myocytes of rats by way of knockdown of calcineurin-related genes.

## 2 Materials and methods

### 2.1 Materials

The Axopatch 700B patch clamp amplifier and Digidata 1322 data converter were from Axon (USA). The Sutter P-97 microelectrode puller was from Sutter (USA) and the BJ-40 glass microelectrode was from Beijing Zhengtianyi Electronics (China). Trypsin and collagenase (type II) were purchased from Sigma (USA). High-glucose Dulbecco’s modified Eagle’s medium (DMEM), premium fetal bovine serum (FBS), phenylephrine (PE), and 5-bromo-2-deoxyuridine (5-BrdU) were purchased from Gibco-BRL (USA). The following antibodies were used: rabbit anti-calcineurin Aβ (CnAβ) antibody (Merck Millipore, Germany), α striated muscle sarcomere actin (α-SCA) antibody (Sigma), horseradish peroxidase-conjugated anti-rabbit antibody (Santa Cruz, USA), rabbit anti-rat Kv4.2 antibody (Abcam, USA), and mouse-derived anti-rat glyceraldehyde-3-phosphate dehydrogenase (GAPDH) antibody (Shanghai Kangcheng Biotech, China). The Genomic DNA Purification Kit was purchased from Fermentas (Canada). The RNeasy Mini Kit was purchased from Qiagen (China). Ad-CnAβshRNA, the gene mediated by the recombinant adenovirus shRNA interference vector for silencing the A subunit β subtype of CaN, and the empty viral vector (null) were prepared by HanBio (Shanghai, China).

### 2.2 Isolation and culture of ventricular myocytes from neonatal rats

One-day-old neonatal Sprague-Dawley rats of clean grade were provided by the Animal Center of Peking University Health Science Center (license number: SYXK [Beijing] 2011-0039). The rats were sacrificed under anesthesia and the animals were fully immersed in 75% ethanol. The ventricle was harvested, the ventricular tissue was cut into small pieces, and the cells were isolated by enzymatic hydrolysis (final concentration of trypsin is 0.1%; the final concentration of type II collagenase is 0.03%). Ventricular myocytes were obtained by differential adherence and 5-BrdU affinity purification. The cells were cultured in high-glucose DMEM containing 10% FBS at 37°C in a 5% CO_2_ incubator. After 48 h, the cells were cultured with serum-free DMEM with high glucose for the next experiment.

**Ethical approval:** The research related to animal use has complied with all the relevant national regulations and institutional policies for the care and use of animals and has been approved by the Ethics Committee of Guizhou Provincial People’s Hospital (Hospital Ethics Review [2012] No. 001).

### 2.3 Identification of ventricular myocytes

Cells were cultured on fibrin-coated glass slides for 48 h and α-SCA was detected by immunofluorescence staining. Neonatal rat cardiac fibroblasts were used as a negative control (Figure A).

### 2.4 Ad-CnAβshRNA sequence screening

**Figure A2:**
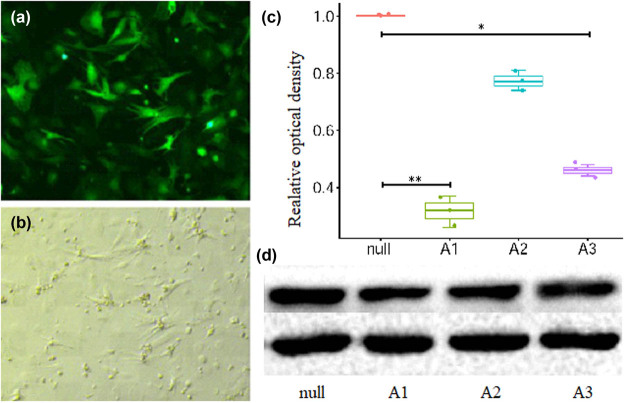
The results of Ad-CnAβshRNA sequence screening. A1 caused the most obvious decrease in the A subunit β subtype of calcineurin (CnAβ) protein expression after its infection of ventricular myocytes for 48 h. (a) Cells appearing green were identified as cells infected by recombinant adenovirus. (b) The photo taken in bright background at the same view of (a). (c) The semiquantitative analysis results of CnAβ protein. *P < 0.05, **P < 0.01; *n* = 3. (d) The representative picture of CnAβ protein detected by western blotting. A1: Ad-CnAβshRNA1. A2: Ad-CnAβshRNA2. A3: Ad-CnAβshRNA3.

Primary ventricular myocytes were cultured for 48 h. Ad-CnAβshRNA1 (A1, interference base sequence 5′-3′: CAGAAAGGGTCTATGAAGCTTGTAT), Ad-CnAβshRNA2 (A2, interference base sequence 5′-3′: CCGCCAGTTTAACTGTTCTCCACAT), Ad-CnAβshRNA3 (A3, interference base sequence 5′-3′: GCAAGATGGCAAGAGTCTTCT), and null at a multiplicity of infection (MOI) of 50 were selected to infect cultured ventricular myocytes for 48 h. CnAβ protein expression in the ventricular myocytes of each group was detected by western blotting. The Ad-CnAβshRNA corresponding to the lowest CnAβ protein expression was regarded as the optimal Ad-CnAβshRNA. A1 caused the most obvious decrease in CnAβ protein expression after its infection of ventricular myocytes at 48 h, and thus it was used in subsequent experiments (Figure A2).

### 2.5 Grouping and interventions

Ventricular myocytes were cultured for 48 h and then cultured in serum-free DMEM. These cells were divided into four groups. (1) In the null group, an adenovirus empty vector at an MOI of 50 was added. After 6 h of infection, the cells were cultured in two volumes of fresh serum-free DMEM for 48 h. (2) In the null + PE group, an adenovirus empty vector at an MOI of 50 was added. After 6 h of infection, the cells were cultured in two volumes of fresh serum-free DMEM with a total of 100 μM of PE (Gibco-BRL, USA) for 48 h. (3) In the A1 group, A1 at an MOI of 50 was added. After 6 h of infection, the cells were cultured in two volumes of fresh serum-free DMEM for 48 h. (4) In the A1 + PE group, A1 at an MOI of 50 was added. After 6 h of infection, the cells were cultured in two volumes of fresh serum-free DMEM with a total of 100 μM of PE for 48 h.

### 2.6 Determining the effectiveness of intervention on hypertrophy in cultured cells

Cell hypertrophy was identified by the measurement of *brain natriuretic peptide (BNP)* mRNA expression and cell size after 48 h of intervention. Upon completion of cell grouping and intervention, real-time reverse transcription-polymerase chain reaction (RT-PCR) was conducted to determine *BNP* mRNA expression in ventricular myocytes. Cells were cultured on glass slides. Crystal violet staining assay was performed after grouping and intervention. Three fields of view were randomly selected in each group. The surface area of the cells was assessed using Image J software.

### 2.7 RT-qPCR

Total RNA was extracted from ventricular myocytes using the RNeasy Mini Kit according to the manufacturer’s instructions. A total of 1 µg of RNA was reverse transcribed using random hexamers from a first-strand cDNA synthesis kit. The mRNAs expression was measured by the real-time PCR method. The cycling conditions were as follows: 95°C for 10 min, followed by 40 cycles of 15 s at 95°C, and 30 s at 60°C. mRNA levels were normalized to GAPDH and determined using the 2-ΔΔCq method. Primers were designed using the Oligo 6.0 software. Specific primers were used to identify and amplify BNP (sense primer: 5′-GTCTCCAGAACAATCCACGA-3′; antisense primer: 5′-CTAAAACAACCTCAGCCCGT-3′), Kv4.2 (sense primer: 5′- GCTCTTCAGCAAGCAAGTTC-3′; antisense primer: 5′- TCCGACTGAAGTTAGACACG-3′), and *GAPDH* (sense primer: 5′- TGATGACATCAAGAAGGTGGTGAAG-3′; antisense primer: 5′- TCCTTGGAGGCCATGTAGGCCAT-3′).

### 2.8 Western blotting

Total protein (40 μg) was loaded and then transferred to a nitrocellulose membrane after electrophoresis. The membrane was blocked with 5% skim milk for 1 h. Rabbit anti-rat GAPDH antibody (1:1,000), rabbit anti-rat rabbit anti-CnAβ antibody (1:500), and rabbit anti-rat rabbit anti-rat Kv4.2 antibody (1:1,000) were added before overnight incubation at 4°C. After thorough rinsing, horseradish peroxidase-labeled secondary antibody was added for incubation at room temperature for 1 h. After thorough rinsing again, color development, photography, and quantitative measurement of the gray scale were performed.

### 2.9 Whole-cell patch clamp detection

A glass microelectrode formed a high resistance seal with the cells and ruptured the membrane. *I*_to_ was recorded under the voltage clamp mode. Current density analysis was used (current density [pA/pF] = current intensity/capacitance) to avoid errors caused by cell size. The action potential of the individual cells was recorded under the current clamp mode. The current signal was guided by an Ag/AgCl electrode, amplified by a patch clamp AXON 700B amplifier through an AD/DA converter board, and stored in a computer hard disk. During the experimental procedure, stimulation discharge and signal acquisition were controlled by pCLAMP 10.0 software.

In the *I*_to_ depolarization step, the clamping voltage was set to −80 mV with an −40 mV to +70 mV pulse stimulation series, with a step voltage of 10 mV, wave width of 300 ms, and frequency of 0.2 Hz. The *I*_to_ steady-state activation curve stimulation protocol was as follows. The clamping voltage was set to −80 mV with a −40 to + 70 mV pulse stimulation series, with a step voltage of 10 mV and a wave width of 300 ms. The *I*_to_ current was then recorded. The *I*_to_ steady-state inactivation curve stimulation protocol was as follows. The clamping voltage was set to −80 mV with a −40 mV to +50 mV pulse stimulation series, with a step voltage of 10 mV and a wave width of 1,000 ms. The residual current was then recorded. The steady-state activation and inactivation curves were created using the normalized current values obtained by the two above-mentioned stimulation schemes as the ordinate and the stimulation pulses under different voltages as the abscissa. The semi-activated voltage (*V*_1/2,act_) and semi-inactivated voltage (*V*_1/2,inact_) were calculated by curve fitting using the Boltzmann equation (*I*/*I*_max_ = 1/{1 + exp[(*V*_1/2_ − *V*_m_)/*k*]}).

The action potential recording method was similar to the voltage clamp mode. After membrane sealing, membrane breaking, and compensation, the recording was switched to the current clamp mode. A 1 nA current pulse was then applied, with a wave width of 2.5 ms, which induced the action potential in the ventricular myocytes. The APDs at 20, 50, and 90% repolarization (APD20, APD50, and APD90) were recorded and analyzed.

### 2.10 Statistical analysis

Statistical analysis was performed using the Graphpad Prism 6 software. All data are expressed as mean ± SD. Differences among groups were compared by one-way analysis of variance, and the *q* test was used for comparison of the two groups. A *P* value of <0.05 was considered statistically significant.

## 3 Results

### 3.1 Effectiveness of stimulation on *BNP* mRNA expression and the surface area of ventricular myocyte

**Figure 1:**
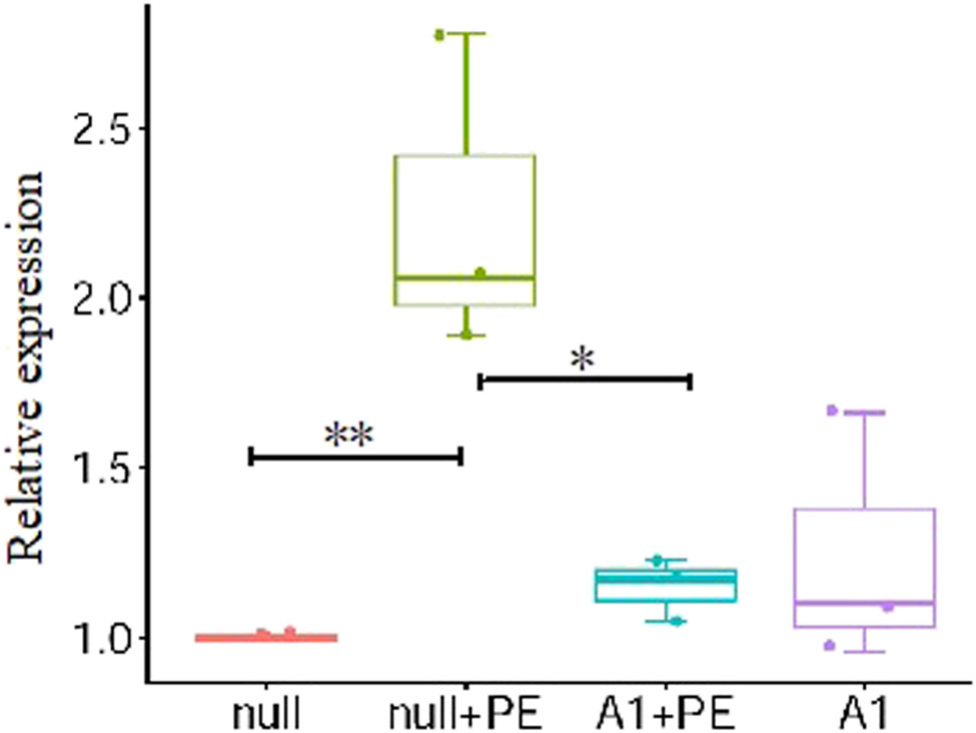
PE stimulation promoted the *BNP* mRNA expression in ventricular myocytes which was attenuated by pre-treatment with Ad-CnAβshRNA1. Bar chart showed the quantitative analysis results of *BNP* mRNA by real-time reverse transcription-polymerase chain reaction. *P < 0.05; *n* = 3. PE: Phenylephrine. A1: Ad-CnAβshRNA.

**Figure 2:**
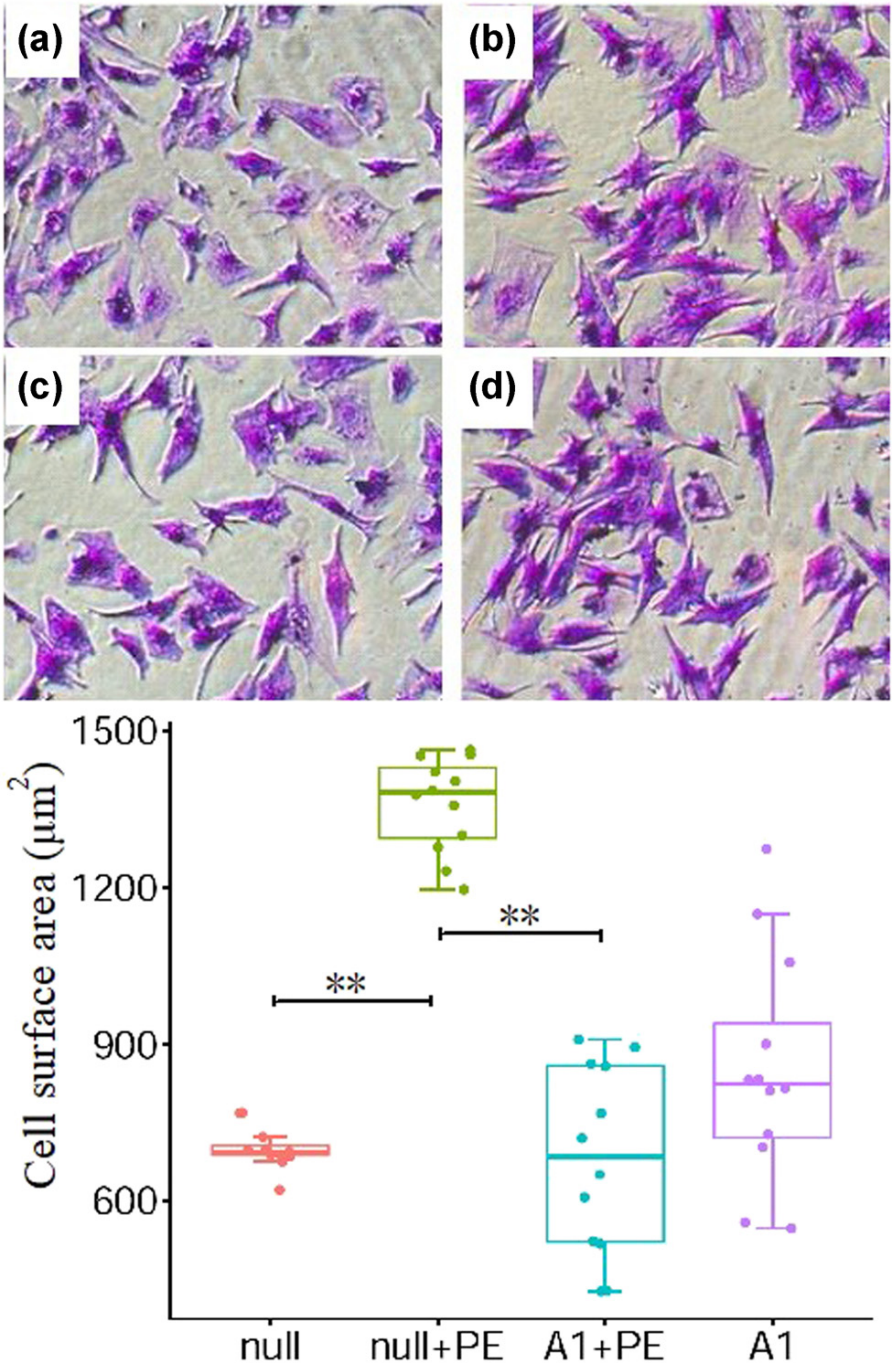
PE stimulation promoted cell hypertrophy which was attenuated by pre-treatment with Ad-CnAβshRNA1. Color picture showed the ventricular cells stained with crystal violet in four groups. a, b, c, and d are the null, null + PE, A1 + PE, and A1 groups, respectively (×100). The histogram showed the comparative results of cell area in groups. *P < 0.05, **P < 0.01. PE: Phenylephrine. A1: Ad-CnAβshRNA.

In the null + PE group, PE treatment for 48 h significantly upregulated *BNP* mRNA expression by an average of 2.24 times (*P* < 0.01), and significantly increased the surface area ((1,360 ± 90) µm^2^ vs (700 ± 40) µm^2^, *P* < 0.01) of ventricular myocytes compared with the null group. Therefore, PE intervention led to hypertrophy of ventricular myocytes. The *BNP* mRNA expression in the A1 + PE group was markedly attenuated compared with that in the null + PE group (1.15 ± 0.09 vs 2.24 ± 0.48, *P* < 0.05), as well as the surface area of cells ((680 ± 180) µm^2^ vs (1,360 ± 90) µm^2^, *P* < 0.01), indicating that the cellular hypertrophy induced by PE was significantly inhibited by Ad-CnAβshRNA intervention (Figures 1 and 2, and Table 1).

**Table 1:** Comparison of the expression of mRNAs and proteins, and the cell area in each group (*x* ± *s*)

	*n*	Null	Null + PE	A1 + PE	A1
*BNP* mRNA	3	1**	2.24 ± 0.48	1.15 ± 0.09*	1.24 ± 0.37
Cell area (µm^2^)	12	700 ± 40**	1360 ± 90	680 ± 180**	850 ± 220
CnAβ protein	3	1**	2.29 ± 0.24	0.90 ± 0.12**	1.18 ± 0.07
*Kv4.2* mRNA	5	1*	0.62 ± 0.07	1.35 ± 0.18**	1.2 ± 0.21
Kv4.2 protein	4	1*	0.58 ± 0.11	0.88 ± 0.09*	1.05 ± 0.2

The expression of mRNAs and proteins were exhibited as relative data. Compared with null + PE group, *P < 0.05, **P < 0.01. PE: phenylephrine. A1: Ad-CnAβshRNA. *BNP*: brain natriuretic peptide. CnAβ: calcineurin Aβ.

### 3.2 Effect of Ad-CnAβshRNA intervention on PE-induced CnAβ protein expression in ventricular myocytes

**Figure 3:**
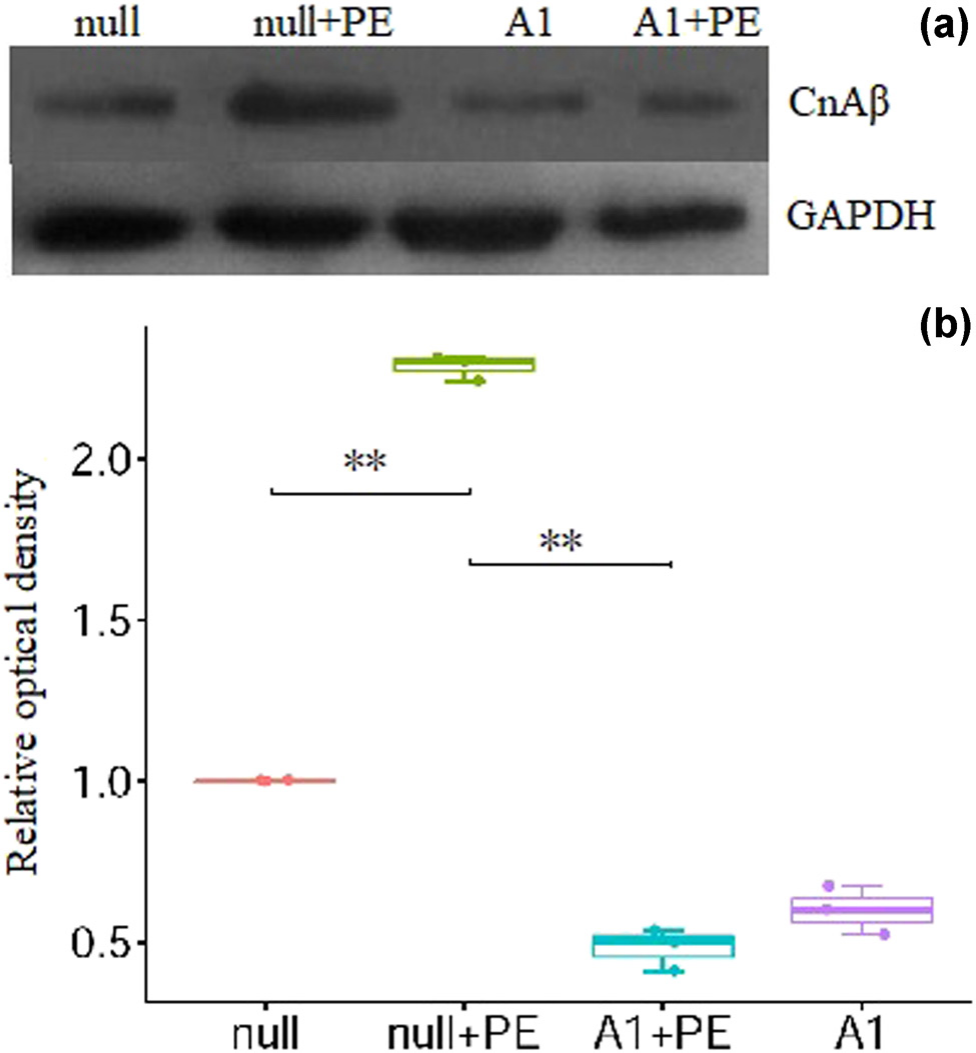
PE stimulation increased CnAβ protein expression which was attenuated by pre-treatment with Ad-CnAβshRNA1. (a) The representative picture of CnAβ protein detected by Western blotting. (b) The semiquantitative analysis results of CnAβ protein in groups. **P < 0.01, *n* = 3. CnAβ: Calcineurin Aβ. PE: Phenylephrine. A1: Ad-CnAβshRNA.

In the null + PE group, PE treatment significantly upregulated CnAβ protein expression by an average of 2.29 times (*P* < 0.01) when compared with the null group. In contrast, CnAβ protein expression was significantly lower in the A1 + PE group than in the null + PE group (0.90 ± 0.12 vs 2.29 ± 0.24, *P* < 0.01) (Figure 3 and Table 1).

### 3.3 Effect of Ad-CnAβshRNA intervention on PE-induced *Kv4.2* mRNA and protein expression in ventricular myocytes

**Figure 4:**
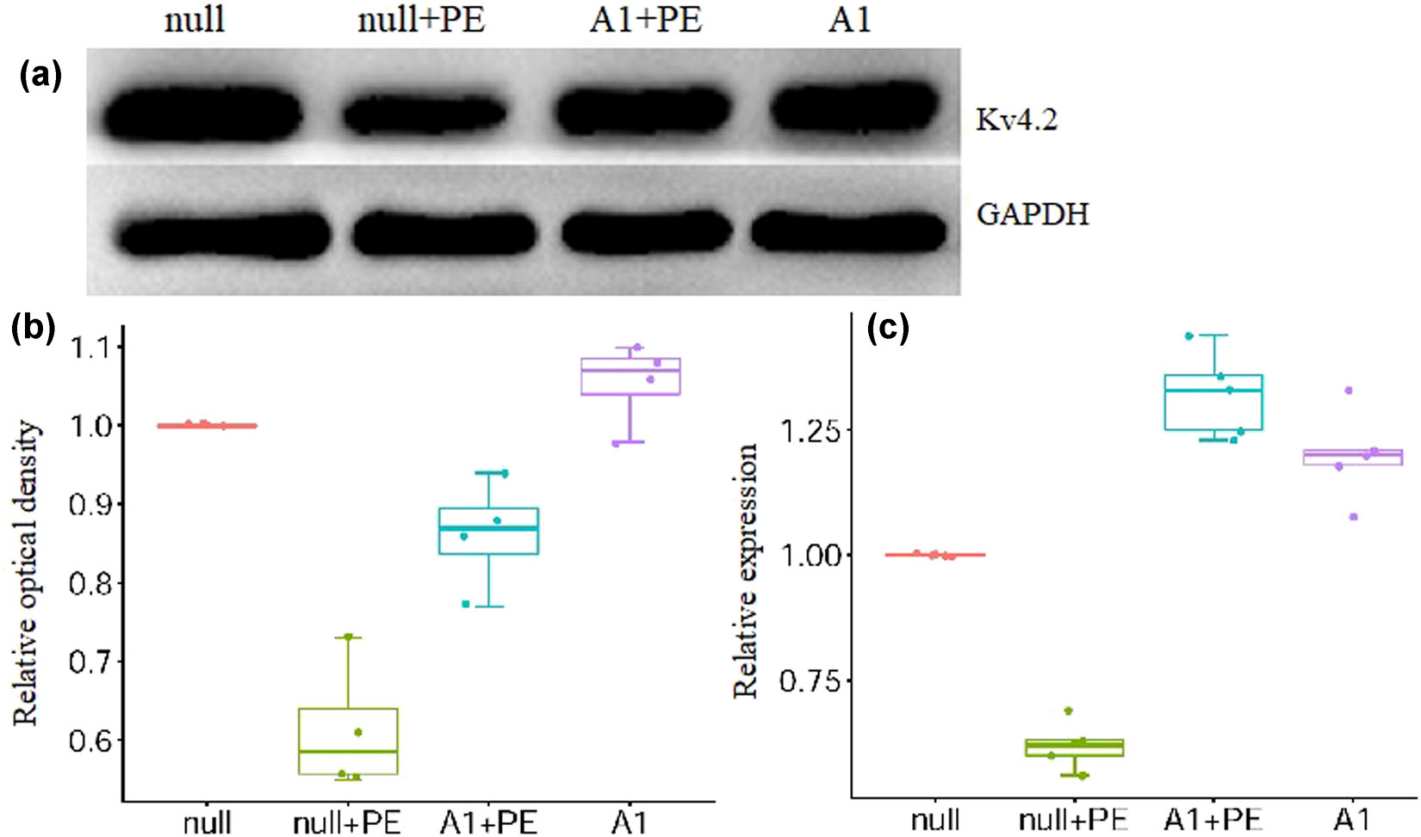
PE intervention inhibited the protein and mRNA expression of Kv4.2 which was attenuated by pre-treatment with Ad-CnAβshRNA1. (a) The representative picture of Kv4.2 protein detected by western blotting. (b and c) The semiquantitative analysis results of Kv4.2 protein (*n* = 4) and mRNA (*n* = 5) in groups, respectively. *P < 0.05, **P < 0.01. PE: Phenylephrine. A1: Ad-CnAβshRNA.

In the null + PE group, PE treatment significantly downregulated *Kv4.2* mRNA (1 vs 0.62 ± 0.07, *P* < 0.05) and protein (1 vs 0.58 ± 0.11, *P* < 0.05) expression when compared to the null group. In contrast, *Kv4.2* mRNA (1.35 ± 0.18 vs 0.62 ± 0.07, *P* < 0.01) and protein (0.88 ± 0.09 vs 0.58 ± 0.11, *P* < 0.05) expression was significantly higher in the A1 + PE group than in the null + PE group (Figure 4 and Table 1).

### 3.4 Effect of Ad-CnAβshRNA intervention on *I*_to_ in ventricular myocytes

**Figure 5:**
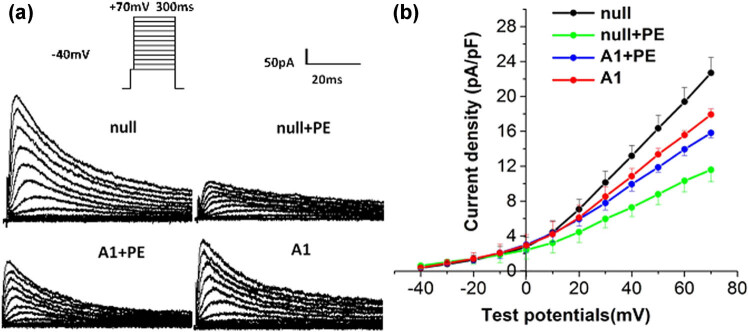
Densities of transient outward potassium current (*I*_to_) in ventricular myocytes. (a) A typical *I*_to_ recording from isolated ventricular myocytes in groups. (b) Current–voltage (*I*–*V*) curve relations. At a stimulation voltage of +20 to +70 mV, PE intervention significantly decreased *I*_to_ density (*P* < 0.05), which was markedly attenuated by pre-treatment with Ad-CnAβshRNA1 (*P* < 0.05; the cell numbers are 14, 12, 12, and 12 in null, null + PE, A1 + PE and A1 groups, respectively). PE: Phenylephrine. A1: Ad-CnAβshRNA.

**Figure 6:**
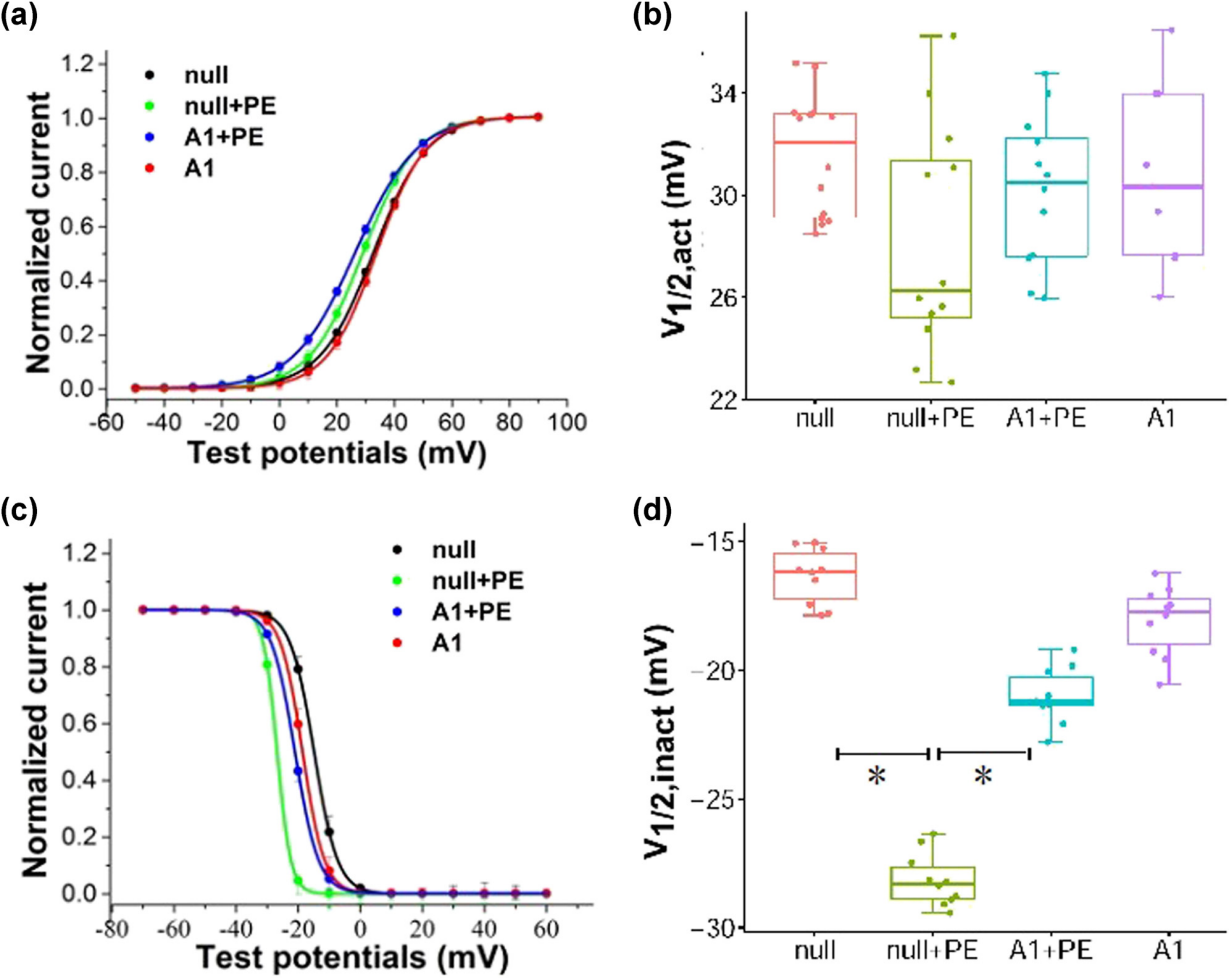
The activation and inactivation curves of transient outward potassium current (*I*_to_). (a) The steady-state activation curve of *I*_to_. (b) The semi-activated voltage (*V*_1/2,act_) of *I*_to_. There were no significant differences of *V*_1/2, act_ among groups (*P* > 0.05; the cell numbers are 14, 12, 12, and 9 in null, null + PE, A1 + PE, and A1 groups, respectively). (c) The steady-state inactivation curve of *I*_to_. (d) The semi-inactivated voltage (*V*_1/2,inact_) of *I*_to_. PE stimulation accelerated inactivation of *I*_to_, which was inhibited by Ad-CnAβshRNA1 intervention (*P < 0.05; *n* = 10 in each group). PE: Phenylephrine. A1: Ad-CnAβshRNA.

At a stimulation voltage of +20 to +70 mV, *I*_to_ current density in the null + PE group was significantly lower than that in the null group (Table 2), the averaged current–voltage (*I*–*V*) curve relations of *I*_to_ remarkably shifted downward, and the peak current density was decreased by 49% (*P* < 0.05; Figure 5). The A1 + PE group had significantly higher *I*_to_ current density than the null + PE group (*P* < 0.05; Figure 5 and Table 2). The shape and distribution of the activation curves of *I*_to_ were similar among the different groups (Figure 6a), and the *V*_1/2,act_ showed no significant difference among the groups (*P* > 0.05; Figure 6b and Table 3). The inactivation curve of *I*_to_ was significantly shifted to the left in the null + PE group when compared with the null group and was significantly shifted to the right in the A1 + PE group compared to the null + PE group (Figure 6c). The *V*_1/2,inact_ was significantly lower in the null + PE group than in the null group (*P* < 0.05) and significantly higher in the A1 + PE group than in the null + PE group (*P* < 0.05; Figure 6d and Table 3). Therefore, stimulation of ventricular myocytes with PE accelerated the inactivation of *I*_to_, whereas Ad-CnAβshRNA1 intervention inhibited such an effect.

**Table 2:** Comparison of current densities (pA/pF) at different voltages in each group (*x* ± *s*)

	*n*	20 mV	30 mV	40 mV	50 mV	60 mV	70 mV
Null	14	7.83 ± 1.04	11.03 ± 1.15	14.28 ± 1.22	17.48 ± 1.45	20.99 ± 1.76	24.02 ± 1.86
Null + PE	12	5.04 ± 1.24	6.27 ± 1.18	7.80 ± 1.21	9.10 ± 1.25	10.89 ± 1.51	12.27 ± 1.88
A1 + PE	12	6.38 ± 0.79	8.45 ± 0.79	10.46 ± 0.88	12.33 ± 1.12	14.65 ± 1.24	16.85 ± 1.38
A1	12	7.41 ± 1.10	9.86 ± 1.06	12.76 ± 0.86	15.38 ± 0.51	18.51 ± 0.64	21.15 ± 0.89

At a stimulation voltage of 20–70 mV: null vs null + PE, *P* < 0.05; A1 + PE vs null + PE, *P* < 0.05. PE: phenylephrine. A1: Ad-CnAβshRNA.

**Table 3:** Comparison of *V*_1/2_ of the activation curves and inactivation curves in each group (*x* ± *s*)

	*n*_act_	*V*_1/2,act_ (mV)	*n*_inact_	*V*_1/2,inact_ (mV)
Null	14	31.57 ± 2.38	10	−16.34 ± 1.07
Null + PE	12	28.21 ± 4.47	10	−28.15 ± 1.00*
A1 + PE	12	30.21 ± 2.91	10	−21.01 ± 1.07
A1	9	30.72 ± 3.51	10	−18.07 ± 1.34

Compared with null group or A1 + PE group, *P < 0.05. PE: phenylephrine.

A1: Ad-CnAβshRNA. *V*_1/2,act_: semi-activated voltage. *V*_1/2, inact_: semi-inactivated voltage.

*n*_act_ and *n*_inact:_ cell number in each group enrolled testing of *V*_1/2,act_ or *V*_1/2,inact._

### 3.5 Effect of Ad-CnAβshRNA intervention on APD in ventricular myocytes

**Figure 7:**
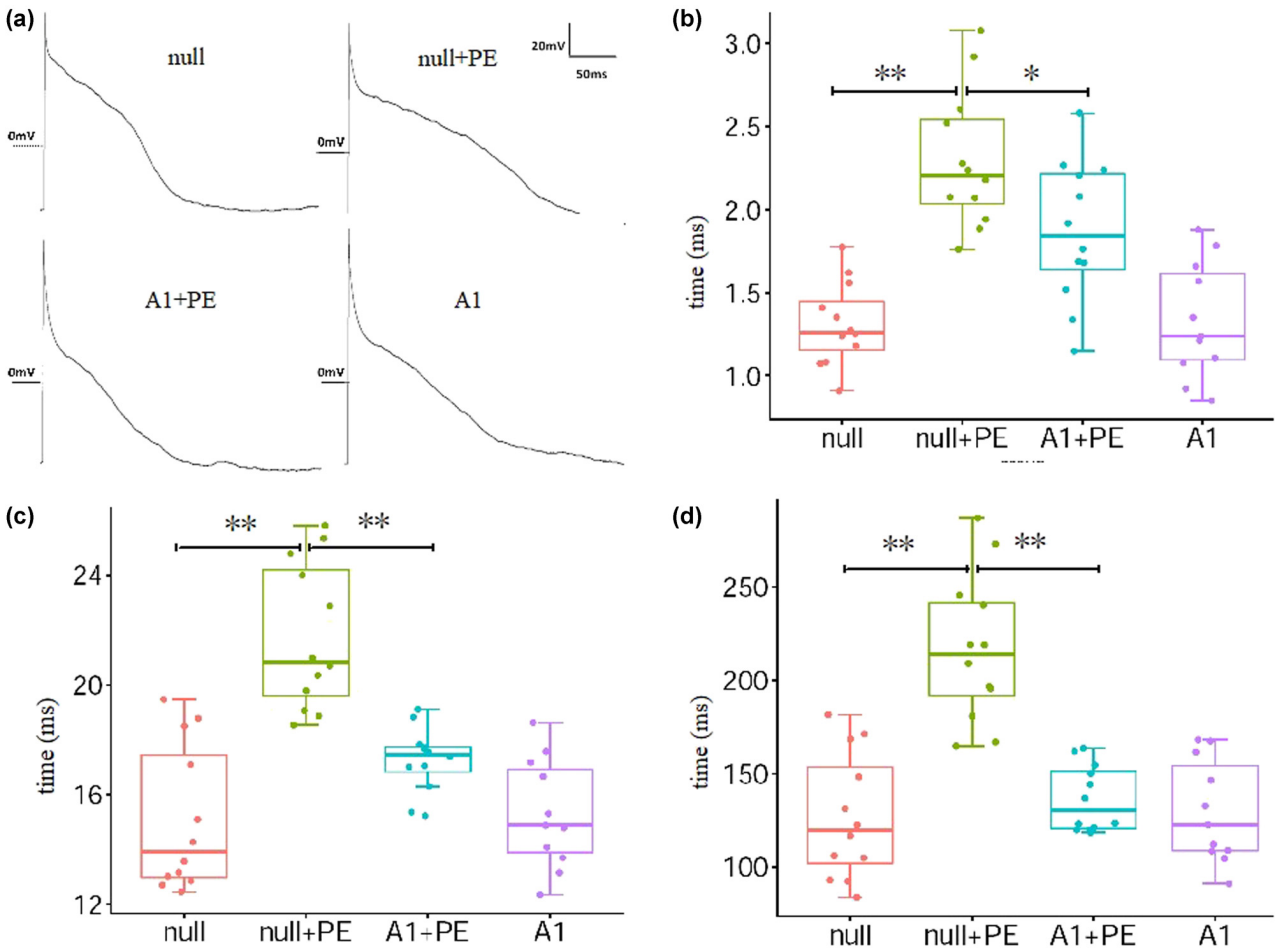
Action potentials of ventricular myocytes. (a) Representative action potentials of cultured ventricular myocytes. (b–d) The analysis results of the APD20, APD50, and APD90 in groups. PE stimulation prolonged the APD, which was inhibited by Ad-CnAβshRNA1 intervention. **P < 0.001, *P < 0.05; the cell numbers are 12, 12, 12, and 11 in null, null + PE, A1 + PE, and A1 groups, respectively. PE: Phenylephrine. A1: Ad-CnAβshRNA.

APD20, APD50, and APD90 were significantly longer in the null + PE group than in the null group and were significantly shorter in the A1 + PE group than in the null + PE group (Figure 7 and Table 4). Therefore, PE intervention led to significant prolongation of APD in ventricular myocytes, whereas Ad-CnAβshRNA intervention attenuated such an effect.

**Table 4:** Comparison of APD (ms) in each group (*x* ± *s*)

	*n*	APD_20_	APD_50_	APD_90_
Null	12	1.31 ± 0.26**	15.08 ± 2.65**	126.66 ± 35.55**
Null + PE	12	2.30 ± 0.41	21.75 ± 3.24	216.44 ± 39.43
A1 + PE	12	1.87 ± 0.42*	17.25 ± 1.19**	136.41 ± 17.66**
A1	11	1.33 ± 0.35	15.30 ± 1.98	129.48 ± 27.51

Compared with null group or A1 + PE group, *P < 0.05, **P < 0.01. PE: phenylephrine, A1: Ad-CnAβshRNA. APD: action potential duration.

## 4 Discussion

The reduction of *I*_to_ slows the repolarization of the action potential in the first phase and reduces the depth of the phase 1 notch, thus affecting the activity of other ion channels. The Kv4.3 channel is expressed in human left ventricular muscle and shows a gradient of protein across the ventricular wall, thus forming electrophysiological transmural heterogeneity [16,17]. In the hypertrophic ventricular myocardium in animal models of cardiac hypertrophy or patients with organic heart disease, especially in patients with co-existing heart failure and myocardial injury, the expression of Kv4.3 and Kv4.2 in ventricular myocytes is downregulated and the activity of *I*_to_ channels is reduced. This leads to abnormal early repolarization, repolarization delay, and APD prolongation, which may easily cause fatal ventricular arrhythmias [8,9,18,19].

The molecular structure of the *I*_to_ ion channel includes a pore-forming α subunit and an auxiliary β subunit. The α subunit of *I*_to_ channel has two functionally distinct components. The fast component, *I*_to,fast_ (*I*_to,f_), for example, recovers from inactivation very rapidly with time constants in the range of 60–100 ms [20,21]. In contrast, the slow component, *I*_to,slow_ (*I*_to,s_), recovers from inactivation slowly with time constants on the order of seconds [22,23]. Usually, *I*_to_ is referred to the *I*_to,fast_. The fast component of α subunit is formed by assembly of Kv4.2 subunits, Kv4.3 subunits, or a combination of the two, which shows the heterogeneity of the species and the different regions of the same heart [23,24,25,26,27].

Selective gene silencing using antisense oligonucleotides (AsODNs) targeted against Kv4.2 and Kv4.3 reduced *I*_to,f_ in cultured rodent ventricular myocytes [28,29]. In rat atrial myocytes, AsODNs targeted against Kv4.2, but not Kv4.3, attenuated *I*_to,f_ [30], whereas in human atrial myocytes, *I*_to,f_ was significantly attenuated by Kv4.3, but not by Kv4.2 and AsODNs [31]. In addition, targeted gene deletion of Kv4.2 in mice eliminates ventricular *I*_to,f_, further revealing the critical role of Kv4.2 in the generation of *I*_to,f_ channels in rodents [32]. So, in this study, Kv4.2 was chosen as the research component of *I*_to_ channel other than Kv4.3.

Ventricular hypertrophy is an effective compensation for chronic heart overload, but eventually develops into congestive heart failure because of decompensation following ventricular remodeling [1,2]. Remodeling of numerous ion channels in ventricular myocytes during this process is an important basis for malignant ventricular arrhythmias. Many cell signaling factors are involved in regulating ion channel remodeling in hypertrophic cardiomyocytes. Promoting *calcineurin* mRNA and protein expression and enhancing calcineurin activity can promote hypertrophy of cardiomyocytes and participate in regulating multiple ion channel remodeling in cardiomyocytes [10,12,13,29]. In rat models of myocardial infarction, the calcineurin inhibitor cyclosporin A can significantly inhibit ventricular remodeling and hypertrophy, improve diastolic function, inhibit a decrease in *I*_to_ current density in ventricular myocytes, and downregulate *Kv4.2* and *Kv4.3* mRNA and protein expression [14]. In ventricular myocytes of mouse myocardial infarction models, *I*_to_ current density is decreased, and *Kv4.2* and *Kv4.3* mRNA and protein expression is downregulated [8]. Furthermore, the β-blocker metoprolol, the calcineurin inhibitor cyclosporin A, and knockout of calcineurin-specific downstream signaling factors (i.e., *NFAT4* gene, a member of the nuclear factor of activated T cells [NFAT] family) can significantly inhibit a decrease in *I*_to_ current density and inhibit changes in *Kv4.2* and *Kv4.3* mRNA and protein expression. In hypertrophic neonatal rat cardiomyocytes, activation of the calcineurin-NFAT signal upregulates the transcriptional expression of *Kv4.2* mRNA and protein and increases *I*_to_ current density [9]. In canine ventricular myocytes with simulated ventricular tachycardia, *Kv4.3* mRNA and protein expression is downregulated, and *I*_to_ current density is significantly reduced, which manifests as significant inhibition of cyclosporine [15].

In this study, the knockdown of the *CnAβ* gene, which encoded the main functional unit of calcineurin, completely inhibited CnAβ protein expression, which defected in the substance base that enables calcineurin to function. Therefore, we evaluated the effect of completely suppressed calcineurin activity on *I*_to_. When the stimulation voltage was between +20 and +70 mV, intervention with the conventional α_1_ adrenergic receptor agonist PE significantly reduced *I*_to_ current density in the ventricular myocytes of neonatal rats. The peak current density decreased by 49%, and the *I*–*V* curves of *I*_to_ remarkably shifted downward. Furthermore, APD20, APD50, and APD90 were significantly prolonged. Knockdown of the *CnAβ* gene significantly inhibited the effect of PE intervention on *I*_to_ current density and APD. Moreover, PE stimulation did not affect the activation of *I*_to_, but PE reduced *I*_to_ current density by accelerating its inactivation. Additionally, the knockdown of the *CnAβ* gene inhibited the effect of PE on *I*_to_ inactivation.

The results of earlier studies showed that the precise role of calcineurin in the regulation of *I*_to_ remains unclear [9,13,14,15]. In those studies, calcineurin activity was promoted by the agonist or transgenic method, and was depressed by cyclosporine [9,13,14,15]. As per our knowledge, this study is the first one to assess the role of calcineurin in the regulation of *I*_to_ by way of calcineurin gene silence. Our results potently showed that calcineurin is a negative regulator of *I*_to_ activity in ventricular myocytes from neonatal rats.

In summary, our findings indicated that *CnAβ* gene knockdown can inhibit PE-induced *I*_to_ channel remodeling and APD prolongation in hypertrophic neonatal rat ventricular myocytes. This finding suggested that calcineurin may be a potential target for the prevention of malignant ventricular arrhythmia in hypertrophic hearts. However, the study had some limitations. Many ion channels are involved in the formation of action potential in ventricular myocytes. However, this study only detected the *I*_to_ ion channel. Moreover, as one of the coding genes of *I*_to_ ion channel, the expression of Kv4.3 was not detected.

## Figures and Tables

**Figure A:**
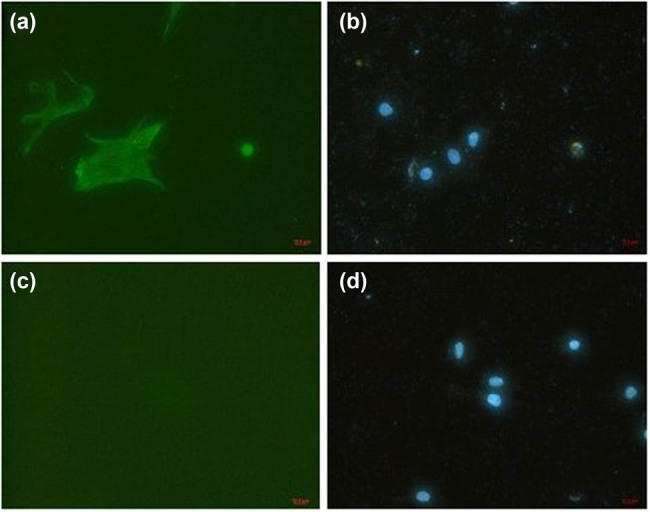
Identification of ventricular myocytes. After 48 h of culture, myocardial cells were demonstrated by immunofluorescent staining with α striated muscle sarcomere actin (α-SCA) antibody that is specialized to striated muscle. (a) Cells appearing green were identified as myocardial cells. (c) Staining for fibroblasts was negative. (b and d) The blue was nuclei stained with diamidine phenylindole. Panels (a) and (b), (c), and (d) are the same view, respectively (×200).
